# The reduction of the thermal quenching effect in laser-excited phosphor converters using highly thermally conductive hBN particles

**DOI:** 10.1038/s41598-021-86249-4

**Published:** 2021-03-24

**Authors:** Akvilė Zabiliūtė-Karaliūnė, Justina Aglinskaitė, Prancis̆kus Vitta

**Affiliations:** Institute of Photonics and Nanotechnology, Faculty of Physics, Vilnius University, Saulėtekio al. 3, 10257 Vilnius, Lithuania

**Keywords:** Materials for devices, Materials for optics, Applied optics, Lasers, LEDs and light sources

## Abstract

Phosphor converters for solid state lighting applications experience a strong thermal stress under high-excitation power densities. The recent interest in laser diode based lighting has made this issue even more severe. This research presents an effective approach to reduce the thermal quenching effect and damage of laser-excited phosphor-silicone converters using thermally conductive hexagonal boron nitride (hBN) particles. Herein, the samples are analyzed by employing phosphor thermometry based on the photoluminescence decay time, and thermo-imaging techniques. The study shows that hBN particle incorporation increases the thermal conductivity of a phosphor-silicone mixture up to 5 times. It turns out, that the addition of hBN to the Eu$^{2+}$ doped chalcogenide-silicone converters can increase the top-limit excitation power density from 60 to 180 W cm$^{-2}$, thus reaching a 2.5 times higher output. Moreover, it is shown that the presence of hBN in Ce$^{3+}$ activated garnet phosphor converters, may increase the output power by up to 1.8 times and that such converters can withstand 218 W cm$^{-2}$ excitation. Besides, hBN particles are also found to enhance the stability of the converters chromaticity and luminous efficacy of radiation. This means that the addition of hBN particles into silicone-based phosphor converter media is applicable in a wide range of different areas, in particular, the ones requiring a high optical power output density.

## Introduction

Solid-state lighting (SSL) based on light-emitting diodes (LEDs) has already occupied a significant part of the lighting market. Due to the constantly increasing efficiency and declining prices, LEDs are widely used for both—general, and niche lighting applications^1,2^. On the other hand, the illumination setups requiring extra high light output power (LOP) density remains a challenge for LEDs due to the exhibited “efficiency droop”^3,4^—the reduction of spontaneous emission efficiency with the increasing electrical power density. In other words, the supply current and resulting light output per square area of the semiconductor are limited and cannot be overcome. Fortunately, the laser diodes (LDs) based on the same family of semiconductors (InGaN) but operated in stimulated light emission regime allow to overcome the limitation of power density and efficiency droop^3–5^. Shuji Nakamura, an inventor of commercial blue/white LEDs and Nobel prize laureate has predicted a bright future for LD based light sources due to the high output power and efficiency per chip size as well as the directionality of the emitted light^6^. Furthermore, several studies comparing LEDs and LDs^3,7,8^ were published and despite the fact that most of them agree on the advantages of LEDs in the low current density levels, LDs are claimed as “exhibiting a clear efficiency advantage over LEDs at higher output power”^8^. LDs have a preference for the intense directional spotlights used for display or projection, entertainment, and architectural illumination^9^. In combinations with precise optics, LDs have already been applied in a few car models (BMW i8, Audi R8 LMX)^10^ and are considered for data transfer applications^11^. However, the implementation of laser light for illumination is not straight forward mostly due to the narrow spectral line and the need to convert the blue beam into the rest of spectral areas of visible light. To employ the advantages of extremely high-power density offered by LDs, the light colour conversion also has to be performed in a tiny space. The blue light conversion fundamentals are the same for LDs and LEDs, where phosphor blends consisting of inorganic phosphor particles embedded within an epoxy or silicone matrix are used for this purpose. The phosphor in the converter absorbs the incident blue light and converts it to the longer wavelength light, eg. yellow^12^. However, the Stokes shift arising from the photon energy difference between the excitation and phosphor photoluminescence (PL), as well as non-ideal quantum yield increase the temperature of the phosphor particles and the entire blend. The heating of the converter might induce the spectral shift and the thermal quenching of the phosphor PL caused by the enhanced non-radiative decay which in turn accounts for an even more rapid converter temperature increase. As a result, the converter might heat up to $540\,^\circ$C (813 K) which can lead to a combustion and permanent damage^13,14^. This effect is even more pronounced with LDs due to the much higher LOP density. It was shown that it takes only 11 s for a silicone based blue phosphor converter to exceed $360\,^\circ$C (633 K) and carbonize when illuminated with a commercial violet LD (595 mW)^15^. This problem can be solved by searching for new thermally robust colour conversion materials. For instance, in the past few years a variety of new phosphors characterized by a particularly high thermal quenching temperatures (between 470 and 600 K) were synthesized^16–21^. As the phosphors per se become less of the problem for the high-power density applications the converter medias consistent of silicone or other organic compounds still remain problematic. To avoid the carbonizing of light converters used for phosphor-converted LDs (pcLD), the high thermal conductivity (TC) materials including but not limited to crystals, glass-ceramics, phosphors in glass (PiG), etc, are proposed^22^. Especially promising results are achieved using phosphor converters made of single-crystals. Víllora et al. and Arjoca et al. have demonstrated that Czochralski grown Y$_3$Al$_5$O$_{10}$:Ce$^{3+}$ (YAG:Ce) and Lu$_3$Al$_5$O$_{12}$:Ce$^{3+}$ (LuAG:Ce) single-crystals are characterized by two orders of magnitude higher TC values if compared to ceramic samples and maintain the QE value above 95% at even $300\,^\circ$C (573 K), whereas for the ceramic powder phosphor it decreases to $\sim$ 82%^23,24^. A thermally stable LuAG:Ce crystal was also successfully grown by a floating zone technique^25^. However, despite the excellent quality of the single crystals, they are characterized by poor light extraction and uniformity. Besides, they have a limited choice of colour temperatures and colour rendering properties due to the lack of phosphor mixtures^22^. Moreover, single-crystal growth process, post-processing complexity and cost, limitation of different materials, and desired concentrations involved, restrict this approach from a wide commercial application. As a result more simple technologies are emerging such as phosphors in glass (PiG) or glass-ceramics^26–29^. PiGs possess such benefits as relatively simple synthesis, low cost, heat resistivity, and durability. Nevertheless, they are characterized by low TC values, and thermal expansion coefficient mismatch between phosphor and glass^22^. Ceramic and glass-ceramic phosphors on the other hand are characterized by high TC values, chemical and physical robustness as well as the ability to control light scattering, absorption, and extraction and are better candidates for pcLD applications^22,30,31^. Currently, several high-quality phosphor ceramics are proposed such as blue BaMgAl$_{10}$O$_{17}$:Eu$^{2+}$ (BAM)^15^, yellow YAG:Ce^9,32,33^, and green LuAG:Ce^34^. However, it is still a great challenge to develop a high-quality broadband red ceramic phosphor since the nitride phosphor ceramics are characterized by a low intrinsic diffusion rate and low relative density^35^.

Another way to increase the TC of the phosphor converter medium is to incorporate the optically transparent composites of high TC. The main advantages of this method are process yield, simplicity, low cost, and applicability for a wide range of powder phosphors. Moreover, it is suitable for both—high power pcLEDs as well as pcLDs and can be easily applied for already existing solid-state lighting architectures. Up to date, the addition of high TC particles to increase the TC value of polymer-based composites was mostly applied for power electronics in order to enhance the thermal dissipation of the devices. For this purpose, polymers are mixed with carbon composites, various metals, nitrides or oxides^36^. However, for optical applications the absorption of visible light would significantly reduce the quantum efficiency (QE) of the device, hence it is of importance to chose a transparent or at least a light-reflecting material in this case. It is known that hexagonal boron nitride (hBN) crystals are transparent for visible light, non-toxic, inert, low-cost, and have a high TC value which is in the range between 180 and 400 W m$^{-1}$ K$^{-1}$^37,38^. hBN powder is characterized by the reflection of light and a significantly wider TC range depending on the crystal plane and varying between 2.5 and 600 W m$^{-1}$ K$^{-1}$^39,40^. However, the powder is more suitable for creating the composites than the crystal as it can be dispersed within binding materials. Besides, hBN powder has already been applied as a thermally conductive filler in polymers for electronic applications^37^. It was shown that the addition of 60 wt% of hBN powder to glass-fiber reinforced-polymer composites increase the TC value from 0.3 to 1.6 W$^{-1}$ K$^{-1}$^41^, and the addition of 88 wt% of hBN to polybenzoxazine resulted in a TC value of the composite as high as 32.5 W m$^{-1}$ K$^{-1}$^42^. Even better results can be achieved if the hBN particles are aligned in the plane direction^40,43^. It was also shown that the addition of hBN particles significantly reduces the thermal quenching of quantum dots used for white LEDs^44^. The increase of TC of polymer materials filled with hBN particles was also confirmed by theoretical models^45,46^. Although hBN containing composites are widely researched for the applications experiencing high thermal flow, the addition of high TC particles is not extensively studied for polymers used for optical applications.

In this paper the effect of hBN particles on the thermal, PL and spectral properties of silicone-based Eu$^{2+}$ activated chalcogenide and YAG:Ce phosphor converters for high power density lighting applications is presented. In order to obtain the most accurate results, two different techniques for the temperature investigation of the phosphor converters are employed. One of them is the temperature monitoring using a thermo-imaging device, which reveals the surface temperature of the converter media. Another, a more comprehensive technique, is based on the PL decay time measurements in frequency domain, and allows to accurately measure the temperature of optically active phosphor particles within the converter^47,48^.

## Results

### PL properties of the phosphor

**Figure 1:**
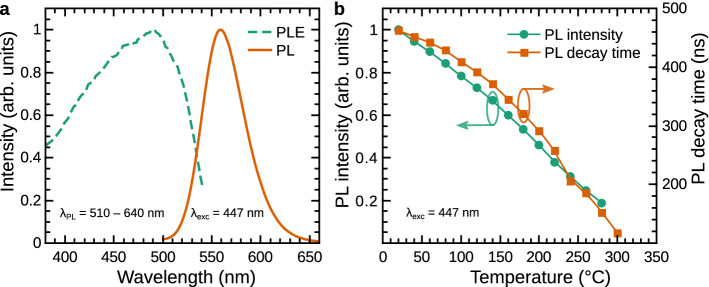
PL properties of the Eu$^{2+}$ activated chalcogenide phosphor BUVY02. (**a**) The PL (solid orange) and PLE (dashed green) spectra. The PLE excitation was evaluated integrating PL spectra between 510 and 640 nm, the PL was recorded under a blue LED excitation (peak wavelength 447 nm). (**b**) The PL intensity (green circles) and PL decay time (orange squares) thermal quenching curves.

Figure 1 presents the PL and PL excitation (PLE) spectra as well as PL intensity and PL decay time thermal quenching curves of a chalcogenide phosphor activated with Eu$^{2+}$ (BUVY02) which was used for the development of light converter in this study^49^. It is seen from Fig. 1a that the PLE takes place in the blue spectral region peaking around 480 nm while the PL consists of a single band in the yellow spectral region with a peak value around 560 nm and a full width at half maximum (FWHM) of around 50 nm. The PL properties confirm the relevance of the BUVY02 phosphor for the lighting applications, since the peak wavelengths of a blue LD (442 nm) or Royal blue LED ($\sim$ 447 nm) emissions are available within the broad PLE band of the phosphor. Figure 1b reveals that the thermal quenching of phosphor PL and PL decay time takes place as soon as the temperature starts to increase. PL intensity and decay time lose 20% of their initial values at approx. 80 and $100\,^\circ$C (353 and 373 K), respectively. This is a particularly low thermal quenching temperature if compared to the commonly used YAG:Ce$^{3+}$ phosphor, which loses 20% of the initial PL intensity and decay time values at roughly 180 and more than $230\,^\circ$C (450 and 500 K), respectively^50^. The pronounced thermal quenching effect of BUVY02 makes it a poor candidate for the high-power density lighting applications. However, this feature is beneficial for the measurements of phosphor temperature in real operating conditions and the investigation of the thermal conductivity properties of light converter media.

### Thermal and PL properties of the phosphor converter containing hBN

**Figure 2:**
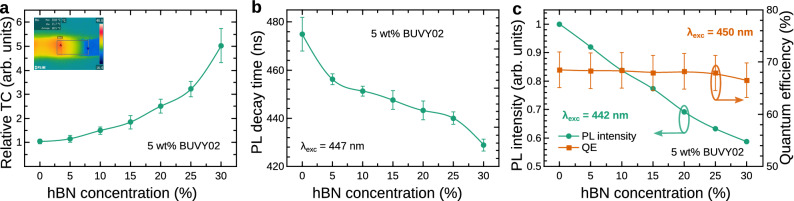
Thermal and PL properties of the phosphor-silicone converter containing 5 wt% BUVY02 phosphor for different hBN concentrations. (**a**) The relative TC of the converter. The inset shows the temperature gradient between the sample and reference recorded during the TC measurements. (**b**) The PL decay time of the converter (measured under 447 nm LED excitation). (**c**) The integrated PL intensity (green circles) and the quantum efficiency (orange squares) of the converter. The PL intensity was measured under 442 nm LD excitation; the PL quantum efficiency was measured under 450 nm excitation.

Figure 2 presents the dependence of the thermal and PL properties of the silicone-phosphor converters containing 5 wt% of BUVY02 phosphor on the hBN concentration in the converter (ranging from 0 to 30 wt%). Figure 2a shows a significant increase of the TC for higher hBN content within the sample. It is clearly seen that for the sample containing 30 wt% of hBN, the TC increases approximately 5 times, if compared to a converter containing barely phosphor. The inset shows the temperature gradient created in the sample and reference during the measurements of TC. Figure 2b shows the PL decay time dependence on the hBN concentration which reveals that for increasing hBN concentration the PL decay time slightly decreases from 475 ± 2 to 430 ± 2 ns, i.e. by $\sim$ 9%. This could be caused by the charge transfer between phosphor and hBN particles which gets stronger as the density of hBN particles increases and the mean distance between them and phosphor particles is reduced. The variation of the PL decay time on the hBN concentration indicates the necessity of PL decay time calibration of all samples, before applying them to phosphor thermometry, in order to get accurate and reproducible results.

**Figure 3:**
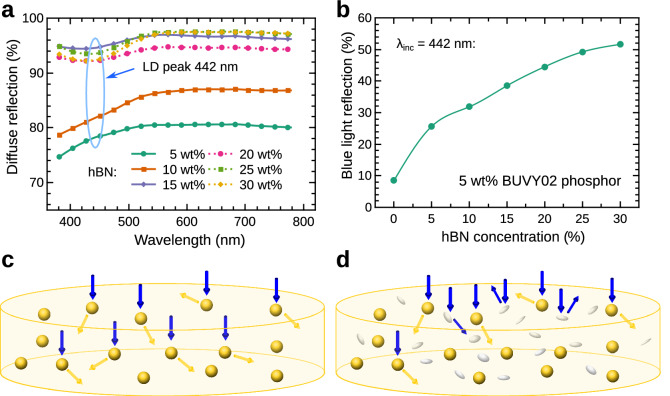
Light reflection mechanism of phosphor-silicone converters. (**a**) The diffuse reflection spectra of silicone converter matrices containing no phosphor for different hBN particle concentrations. The blue ellipse highlights the LD emission peak wavelength. (**b**) The dependence of the LD emission light (442 nm) reflection on the hBN concentration in the phosphor-silicone converter containing 5 wt% of BUVY02 phosphor. (**c**) and (**d**) The excitation of phosphor particles (yellow spheres) in the converter in the absence and presence of hBN particles (white ellipsoids), respectively. Blue arrows correspond to the excitation light, and yellow ones to the phosphor PL.

The dependences of the PL intensity (green) and PL QE (orange) on the hBN concentration are presented in Fig. 2c. Here we can observe a distinctive tendency of the decreasing PL intensity and almost constant PL QE with an increasing hBN concentration. Such behaviour indicates, that the sample absorption rate decreases with increasing content of hBN. In other words, the diffuse reflection (scattering) of the incident light by hBN particles residing in the converter takes place and is explained in Fig. 3. Figure 3a presents the diffuse reflection spectra of the silicone converter matrix containing only different amounts of hBN and no phosphor. The observed increase of the diffuse reflection for higher hBN powder concentration in the sample can be explained by the scattering of light caused by hBN particles which prevents the deeper light penetration into the sample. The blue LD (442 nm) light reflection by the converter containing 5 wt% of BUVY02 phosphor powder for different hBN concentrations is shown in Fig. 3b. Here we can see that the reflectivity of blue light increases from less than 10 to nearly 50% when the concentration of hBN is increased from 0 to 30 wt%. Therefore, the probable propagation of light in the converter is presented in Fig. 3c,d. Figure 3c shows the schematic representation of the silicone phosphor converter containing phosphor particles (yellow spheres) with no hBN powder. Here, the most of the incident photons (blue arrows) reach the target, are absorbed, and converted to yellow light (yellow arrows) by phosphor particles. However, the addition of hBN particles (Fig. 3d, white ellipsoids) increases the scattering of the incident light which causes it to reflect backwards without being absorbed by phosphor particles lying deeper in the sample. This leads to a distinctive decrease of the PL intensity, yet keeping an almost constant QE. To be accurate enough, we have to mention that the QE shows a slight decrease (within the uncertainty of the measurements) which is in line with the decrease of PL decay time (Fig. 2b) with hBN concentration increase. Nevertheless, it can be summarized that hBN inclusions act as spectrally neutral light scattering particles and are responsible for only a minor quenching of the PL decay ($\sim$ 9%).

**Figure 4:**
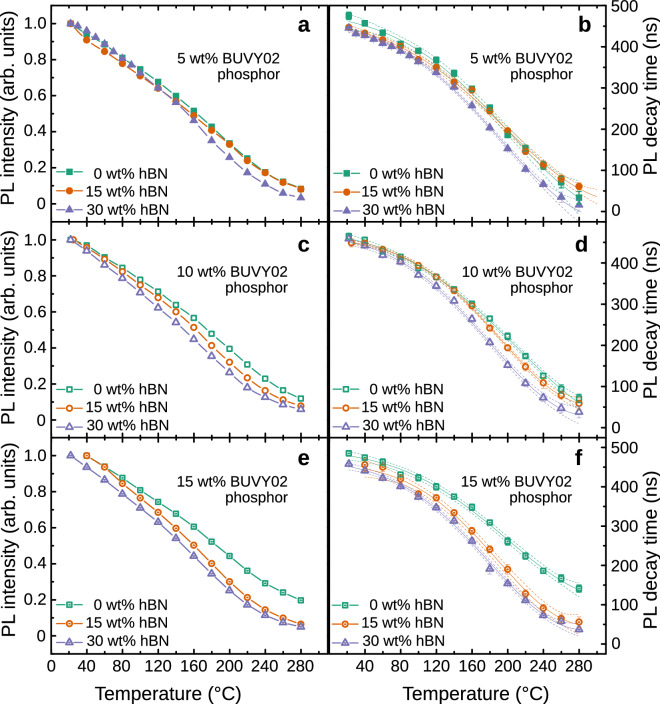
Calibration curves used for the phosphor thermometry. (**a**), (**c**), and (**e**) present the dependence of the PL intensity of the converters for different hBN concentrations containing 5, 10 and 15 wt% of BUVY02 phosphor, respectively. (**b**), (**d**), and (**f**) present the PL decay time values of the converters for different hBN concentrations containing 5, 10 and 15 wt% of BUVY02 phosphor, respectively. Solid lines represent the least square fits to the experimental data and the dashed lines show the 95% confidence intervals.

In order to perform accurate temperature measurements and the investigation of thermal properties of light converters containing both, phosphors and hBN at different concentrations, the PL decay time dependencies on temperature had to be calibrated. The calibration curves showing the PL intensity and PL decay time of the converters with different concentrations of BUVY02 phosphor (5, 10, and 15 wt%) and hBN particles (0, 15, 30 wt%) as functions of the sample temperature are provided in Fig. 4a,c,e and b,d,f, respectively. All graphs show a steady decrease of PL intensity and PL decay time with temperature. The PL intensity curves presented in Fig. 4a,c,e demonstrate that the thermal quenching takes place as soon as the temperature increases above room temperature. We can see that the PL intensity drops by 20% at around 70–80$\,^\circ$C and the PL intensity slope shape is qualitatively the same for all samples despite the phosphor concentration in the converter. However, the converters with the highest hBN concentration are characterized by a slightly stronger thermal quenching as this effect seems to increase with hBN concentration. Figure 4b,d,f reveals that the PL decay time dependence on the temperature curve is of a similar shape as that of the intensity. It is seen that the decay time begins to decrease already slightly above the room temperature and reaches 80% of its initial value at around 120$\,^\circ$C. As in the case of PL intensity dependence, we can see that the addition of hBN particles results in a slightly stronger quenching which is mostly pronounced for the converters containing 15 wt% of phosphor concentration. This is probably caused by the interaction between phosphor and hBN particles by means of charge transfer which depends on the mean distance between particles and the temperature helping the carriers to overcome the potential barrier. Solid lines on the PL decay graphs present the fitted curve of the exponential decay law used for the least square error interpolation of the experimental points. These curves were employed for the temperature estimation from the PL decay time in the subsequent measurements. The dashed lines present 95% confidence intervals. The strong dependence of PL properties on the ambient temperature makes this phosphor particularly suitable for phosphor thermometry applications, providing value in the range of 0.5–2.5 ns K$^{-1}$, when PL decay time measurement uncertainty was ± 2%.

### Optical thermometry under LD excitation

**Figure 5:**
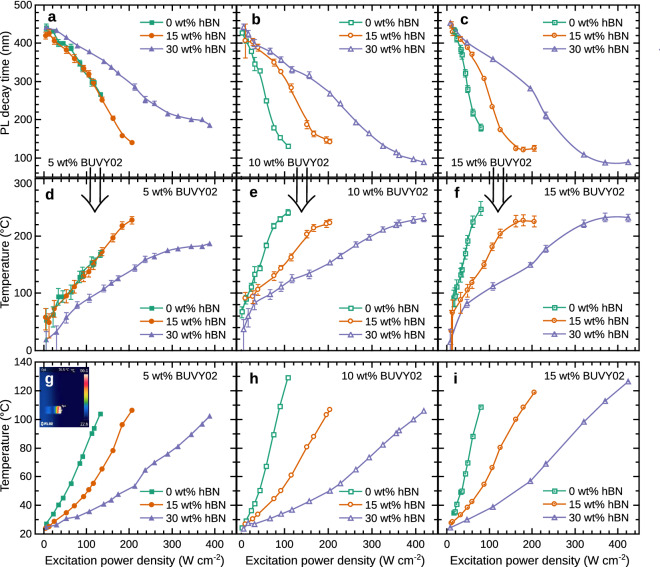
Parameters of the phosphor converters for different LD excitation power densities. (**a**–**c**) The decay time values as functions of LD excitation power density for the converters with different amounts of hBN (0 wt% green, 15 wt% orange, 30 wt% purple) and containing 5, 10 and 15 wt% of BUVY02 phosphor respectively. (**d**–**f**) The temperatures of the samples, extracted from the calibration curves, as functions of LD excitation power density for the converters with different amounts of hBN (0 wt% green, 15 wt% orange, 30 wt% purple) and containing 5, 10 and 15 wt% of BUVY02 phosphor, respectively. (**g**–**i**) The temperatures of the samples measured using a thermovisor as functions of LD excitation power density for the converters with different amounts of hBN (0 wt% green, 15 wt% orange, 30 wt% purple) and containing 5, 10 and 15 wt% of phosphor, respectively. The inset of Figure (**g**) shows a thermographic image of the sample under LD excitation.

Figure 5 shows the PL decay times and temperatures as functions of excitation power density, for the phosphor converters containing 5, 10 and 15 wt% of BUVY02 and 0, 15 and 30 wt% of hBN particles upon blue LD excitation (peak 442 nm). Figure 5a–c show that the PL decay time decreases slightly faster for the samples containing higher phosphor concentrations. This happens since more luminescence centers generate more heat due to the losses arising from the Stokes-shift and non-radiative recombination. It is also seen, that the PL decay value drop is less significant for the samples having higher concentrations of hBN particles, meaning that the increased TC value of the converter matrix helps to reduce the thermal quenching effect. The temperatures of BUVY02 phosphor converters calculated from Fig. 5a–c using the calibration curves shown in Fig. 4b,d,f are presented in Fig. 5d–f, whereas the temperatures measured using a thermo-imaging device are shown in Fig. 5g–i. The inset of Fig. 5g shows a thermographic image of the sample under LD excitation. As expected, the temperature of the converters rises for the increasing excitation power density. However, if we look at the values, we observe that the temperatures recorded by a thermo-imaging device are significantly lower than those derived from the PL decay and the estimated uncertainty bars are too small to diminish such a prominent difference. The main reason for this discrepancy is the fact that the PL decay reflects the temperature of the luminescence centers from within the sample, while the thermovisor shows the averaged temperature of a significantly cooler surface. Similar conclusions were also presented in another research that studied the heating mechanisms and patterns in the phosphor converters for LEDs^47^. Despite the discrepancy of the temperature values, both methods clearly show that the increasing hBN concentration significantly reduces the temperature of the sample. From Fig. 5d–f it is seen that the samples containing 15 wt% of phosphor and no hBN reaches 240 $^\circ$ C at the excitation power density less than 100 W cm$^{-2}$, whereas the sample with the same phosphor concentration containing 30 wt% of hBN reaches the same temperature at around 350 W cm$^{-2}$. This observation confirms that the incorporation of high TC hBN particles to the light converter matrix shifts the thermal quenching threshold to higher excitation power densities.

### Light output measurements

**Figure 6:**
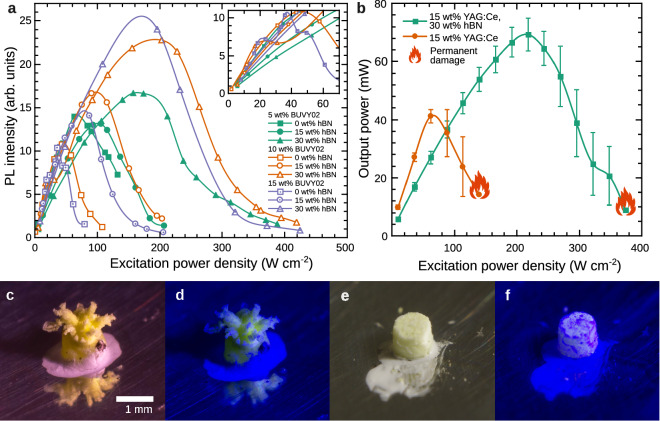
Impact of high power density LD excitation light (peak 442 nm, focus diameter 0.5 mm) on silicone-phosphor converters. (**a**) The PL intensity dependence of different composition 2 $\times$ 2 mm cylinder BUVY02 phosphor-silicone converters on the excitation power density. The inset shows the enlarged area between 0 and 70 W cm$^{-2}$ excitation power density. (**b**) The spectral power emitted by a 1 $\times$ 1 mm cylinder silicone light converter containing only 15 wt% of YAG:Ce phosphor (orange circles) and with additional 30 wt% of hBN powder (green squares) as a function of excitation power density. (**c**,**d**) The physical damage of the silicone light converter containing 15 wt% YAG:Ce phosphor caused by the LD excitation of 160 mW optical power (88 W cm$^{-2}$) under white (conventional fluorescent lamp) and blue 447 nm LED light respectively. (**e**,**f**) An analogous converter additionally containing 30 wt% of hBN particles affected by 400 mW LD excitation (218 W cm$^{-2}$). In the latter case no physical damage is observed.

As mentioned before, the high concentration of hBN particles besides increasing the TC also increases the reflection and scattering of the excitation light limiting the effective density of the absorbed power. In order to find out if the temperature reduction caused by the increase in TC of the converter media is more significant than due to the absorption decrease, the spatially integrated PL intensity value of different converters as a function of LD excitation power density was measured. These results for BUVY02 phosphor silicone converters containing 5, 10 and 15 wt% of phosphor and 0, 15 and 30 wt% of hBN are presented in Fig. 6a. The symbols mark the measured points while the line is a spline fit depicted as a guide for the eye. The inset in the Figure shows the enlarged view in the excitation power density range between 0 and 70 W cm$^{-2}$. It is seen in the Figure, that for pure samples, containing no hBN particles, the maximum PL intensity value is observed for the sample with the lowest phosphor concentration. This arises due to the stronger thermal quenching effect, since more luminescence centres generate more heat. It is also observed that for low excitation densities the PL intensity increases faster and is higher for the converters with no hBN particles. However, at some level of the excitation density, the samples with hBN overcome the pure ones. As it is observed in Fig. 5 the rising excitation density increases the temperature of the converter which causes the PL quenching. This process is slower in the converters containing hBN filler of high TC. As a result, the peak value of the PL intensity of the converter containing 15 wt% of phosphor and 30 wt% of hBN powder is around 2.5 times higher than for the one with no hBN particles.

Since the BUVY02 phosphor is characterized by a strong thermal quenching, it is perfectly suitable for phosphor thermometry or the demonstration of the positive thermally conductive filler effect within the phosphor converter. However, it is not well suited for the practical lighting applications due to the high sensitivity to the rising temperature. For commercial high-power applications, phosphors of the highest possible quenching temperatures and thermal stability are required. In order to test the applicability of hBN together with high thermal stability phosphors like YAG:Ce, the silicone-phosphor converters of tight dimensions (1 $\times$ 1 mm cylinder) containing YAG:Ce$^{3+}$ and hBN particles were developed, and the total output power was measured inside an integrating sphere.

Figure 6b shows the LOP values (the averages of 7 samples of each type) of YAG:Ce$^{3+}$ 15 wt% silicone converters (orange) and the converters with additional 30 wt% of hBN powder (green). One can observe a similar tendency as in the case of BUVY02 phosphor. Under low excitation, LOP is higher for the converters containing barely phosphor. However, the rising excitation power increases the temperature of the phosphor and thermal quenching takes place. As a result, the PL intensity of the converter with no hBN particles starts to decrease, yet the PL intensity of the converter containing thermally conductive hBN particles keeps rising. The peak value of LOP for the sample without hBN is around 41 mW at approximately 60 W cm$^{-2}$ excitation density, meanwhile for the one containing hBN filler is almost 1.8 times higher and reaches around 70 mW under approximately 218 W cm$^{-2}$ excitation power density.

The orange flames in Fig. 6b represent the excitation level at which the majority of the samples have experienced a permanent damage. This value was around 140 and 374 W cm$^{-2}$ for the converters without and with 30 wt% of hBN particles, respectively. 374 W cm$^{-2}$ is a good result for silicone, since it is around half of the value that a high quality ceramic phosphor converters can withstand^9^. It is important to note, that the exact level of excitation resulting the permanent damage was rather sample dependent and caused a significant uncertainty for these measurements, which are also seen in Fig. 6b. The image of the damaged YAG:Ce$^{3+}$ phosphor-silicone converter with no hBN powder is presented in Fig. 6c,d, under white (4000 K) and blue (445 nm) LED illumination, respectively. It is seen that for most of the samples the high LD excitation power density (88 W cm$^{-2}$) caused the burst of the phosphor converter to a hydra-like shape rather than burnt it. The phosphor converter containing hBN particles affected by an even higher excitation power density (218 W cm$^{-2}$) under the same lighting conditions is presented in Fig. 6e,f, respectively. In this case no physical damage is observed. Moreover, under the blue light illumination, hBN particle clusters are revealed as dark blue and purple patches.

**Figure 7:**
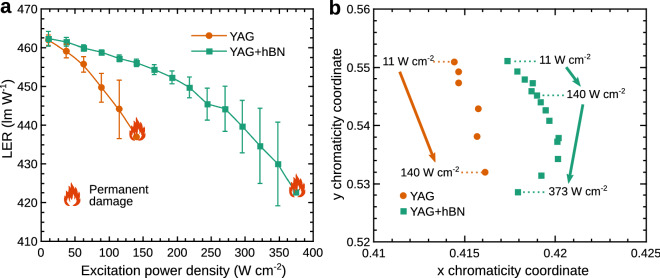
Spectral parameters of 15 wt% YAG and 15 wt% YAG + 30 wt% hBN silicone converters for different excitation power densities. (**a**) The dependence of LER on the excitation power density. (**b**) The xy chromaticity coordinates for different excitation power densities.

Figure 7 shows the spectral parameters of 15 wt% YAG and 15 wt% YAG + 30 wt% hBN silicone converters for different excitation power densities. Figure 7a shows the dependence of the luminous efficacy of radiation (LER) on the excitation power density. We can see, that for low excitations (11 W cm$^{-2}$) LER is the same for both converters and is equal to 462 lm W$^{-1}$. However, for higher excitation power densities the temperatures of the converters increase, and this increase is more rapid in a converter with no hBN particles. The rising temperature red-shifts the PL spectrum of the phosphor thus reducing the LER. This finding is also supported by Fig. 7b which presents CIE xy chromaticity coordinates of the converters for different excitation power densities. Again, we can see, that the converter containing barely YAG phosphor is affected much more, and has a more pronounced change of the chromaticity.

## Discussion

To sum up, a new approach to reduce the thermal quenching and the threshold of the physical damage of phosphor converters for high power LEDs and LDs was presented. It was shown that by using a simple and relatively low-cost material like hBN powder, the TC of silicone matrix used for phosphor converters, can be increased five times. Despite the adverse effects caused by the large concentrations of hBN particles, such as the reflection and scattering of the excitation light or a slight decrease of the QE of the phosphor, the over-all PL intensity and LOP measurements for different LD excitation power densities have demonstrated a clear benefit of hBN particle incorporation for high excitation power densities. For a 15 wt% chalcogenide phosphor-silicone converter characterized by a particularly low thermal quenching threshold the maximum PL intensity was increased by around 2.5 times and peaked at the excitation densities from 60 to 180 W cm$^{-2}$ depending on the concentration of hBN powder. Furthermore, similar results were obtained for a widely used YAG:Ce$^{3+}$ phosphor. The optical output power for 15 wt% YAG:Ce$^{3+}$ phosphor-silicone converter was by 1.8 times larger when 30 wt% of hBN powder was present, and the excitation power has shifted from 60 to nearly 220 W cm$^{-2}$. Moreover, it was shown, that the addition of hBN particles has a positive effect on the spectral parameters of YAG phosphor converters as it weakens the dependence of LER and chromaticity on the excitation power density. This approach was shown to be effective for two kinds of phosphors characterized by different physical properties and it may be suitable for a vast variety of other materials that should be tested in the future. The temperature decrease of the phosphor converter could be extremely beneficial when mixtures of different phosphors are employed and suffer from colour change due to the different thermal quenching properties. The hBN incorporation can be easily realized in already existing technological processes, lighting designs and set-ups. For this reason, it could be applied in a wide range of different areas, in particular, the ones requiring a high optical power output density: e.g. car headlights, illumination of buildings or mass events as well as general lighting of large spaces.

## Methods

### Preparation of the samples

The cylinder shaped chalcogenide (BUVY02, PhosphorTech, chemical formula Ca$_{\text {w}}$Sr$_{\text {x}}$Ga$_{\text {y}}$(S,Se)$_{\text {z}}$:Eu$^{2+}$)^49^ phosphor-silicone converters were prepared in two different sizes: 3 mm thickness and 12 mm diameter as well as 2 mm thickness and 2 mm diameter, for the TC and spectroscopic measurements, respectively. The cylinder shaped garnet (HTY550 PhosphorTech, general chemical formula Y$_3$Al$_5$O$_{12}$:Ce$^{3+}$, YAG:Ce) phosphor converters were prepared in one size: 1 mm thickness and 1 mm diameter.

**Table 1:** Dimensions and composition of the samples, as well as the performed measurements.

Phosphor	Sample size	Phosphor concentration	hBN concentration	Measurements
BUVY02	3 $\times$ 12 mm cylinder	5 wt%	0, 5, 10, 15, 20, 25, 30 wt%	TC, QE, PL decay, diffuse reflection
	2 $\times$ 2 mm cylinder	5, 10, 15 wt%	0, 15, 30 wt%	Calibration, PL decay, response to the excitation power density
YAG:Ce	1 $\times$ 1 mm cylinder	15 wt%	0, 30 wt%	LOP

The samples were prepared by mixing phosphor powder and hBN particles (Acros Organics, 98% purity) with a transparent silicone sealant (VersaChem) and pouring to the prepared moulds. BUVY02 phosphor converters contained 5, 10, and 15 wt% of phosphor as well as hBN powder in a range of 0–30 wt%. YAG:Ce phosphor converters contained 15 wt% of phosphor as well as 0 and 30 wt% of hBN powder. The sample compositions are summarized in Table 1. No solvent was applied to avoid any possible chemical interaction with the materials.

### Measurements of the thermal conductivity

The TC for the samples was measured in relative units using the same shape polymethyl methacrylate (PMMA) pellet (TC 0.19 W m$^{-1}$ K$^{-1}$) as a reference. The experimental set-up and its explanation are presented in Supplementary Fig. S1 in the Supplementary Information. The temperature gradients of the reference and the sample were recorded using a thermovisor (FLIR Systems, ThermoVision A320). Overall, five measurements were performed for each sample and the relative TC was calculated as an average value of the sample to reference temperature gradients’ slopes ratios^51^.

### Measurements of the PL properties

The PL QE was measured using an integrating sphere (Sphere Optics, Spectralon white standard) method^52^, the PL signal was registered with Hamamatsu PMA-11 spectrometer. The samples were excited with a xenon lamp light passing through a monochromator set at 450 nm.

The diffuse reflection spectra were measured using AvaLight-HAL-Mini halogen lamp (Avantes) as an excitation source, AvaSphere-50-LS-HAL-CAL sphere (Avantes) and AvaSpec-ULS2048LTEC spectrometer (Avantes). Spectralon (Labsphere) white standard was used as a reference. For the reflection of the excitation light and PL intensity measurements, PLTB450b LD (Osram, peak wavelength 442 nm, 1.4 W) was used as an excitation source.

### PL decay time measurements

The PL decay times of the samples were measured using a frequency-domain (FD) technique^48,53^. This method exhibited a unique advantage in a current study allowing the same source of excitation to be employed for both—heating the sample and exciting the PL. The experimental set-up is presented in Supplementary Fig. S2 in Supplementary Information. The blue LED (Lumileds LUXEON LXML-PR02, peak wavelength 447 nm) is sinusoidally modulated in the 1 kHz–250 kHz frequency range by the signal generator (Tektronix AFG3252) controlled with a computer via the GPIB port and focused to the phosphor sample using two lenses. The short pass filter (cut-off 470 nm) is used in order to filter the excitation signal and make sure that it does not overlap with the PL. The long-pass filters (cut-off 510 nm) were used in order to filter the PL signal and to separate it from the excitation light. The PL signal is registered with a photomultiplier (Hamamatsu, H6780-01) and sent to the lock-in amplifier (Signal Recovery 7265) which measures the AC part of the signal, and the phase shift between PL and excitation LED. It is worth mentioning, that using the FD technique, the PL decay time can be measured from a single modulation frequency point, and the frequency sweep was used only to increase the accuracy and to double-check that single-exponent decay law was correct at all measurement conditions.

During the measurements of the PL decay time dependence on the temperature, the sample was well attached to a copper plate mounted in the cryostat (Cryo Industries). The vacuum was maintained with a rotational pump (Leybold, TRIVAC B D4B) and the temperature was set in the range of 20–280 $^\circ$C using a cryogenic temperature controller (Cryogenic Control Systems 32).

### Measurements of the PL properties on the excitation power density

The experimental set-up of this measurement is presented in Supplementary Fig. S3 in the Supplementary Information. In general, the set-up is very similar to the one used for the PL decay time measurements except for a few differences: a blue 1600 mW LD (Osram PLTB450b, peak wavelength 442 nm) was used as an excitation source instead of the LED; the excitation power was measured using an optical power meter (Ophir Nova), which was calibrated to measure the reflection from the glass plate placed in front of the lens focusing the excitation light; the excitation optical power density was controlled by placing a cuvette filled with water and a variable amount of ink right after the excitation source; a thermo-imaging device (FLIR Systems, ThermoVision A320) was pointed towards the sample in order to measure its surface temperature.

The LOP, LER and xy chromaticity coordinates as functions of excitation power density for YAG:Ce phosphor converters were measured in an integrating sphere (Labsphere, Illumia Pro 500). 1 mm thickness and diameter cylinder shaped converter was attached to a concave mirror using a thermal paste and placed inside the integrating sphere in 2$\pi$ configuration. The LD beam was focused to the sample with the spot size of 0.5 mm. In order to ensure the uniform temperature of the sample at the LD excitation light, there was a 10 min delay each time after changing the excitation power. The SPD of the converter was measured for LD driving currents from 200 to 1000 mA (or until the permanent damage) every 50 mA which corresponds to around 10–425 W cm$^{-2}$ power density range. The LD was calibrated before and after the measurement’s session using an optical power meter (Ophir Nova). Overall, 7 identical samples containing 15 wt% of YAG:Ce and 7 samples containing 15 wt% of YAG:Ce as well as 30 wt% of hBN were examined. The images of the samples were taken using Pentax K-r camera equipped with a macro lens.

## Supplementary Information

Supplementary Information.

## References

[1] Pust P, Schmidt PJ, Schnick W (2015). A revolution in lighting. Nat. Mater..

[2] Cho J, Park JH, Kim JK, Schubert EF (2017). White light-emitting diodes: History, progress, and future. Laser Photon. Rev..

[3] Wierer JJ, Tsao JY, Sizov DS (2013). Comparison between blue lasers and light-emitting diodes for future solid-state lighting: Comparison between blue lasers and light-emitting diodes. Laser Photon. Rev..

[4] Cho J, Schubert EF, Kim JK (2013). Efficiency droop in light-emitting diodes: Challenges and countermeasures. Laser Photon. Rev..

[5] Neumann A (2011). Four-color laser white illuminant demonstrating high color-rendering quality. Opt. Express.

[6] Nakamura S (2015). Background story of the invention of efficient blue InGaN light emitting diodes (Nobel Lecture): Invention of the efficient blue InGaN LEDs. Ann. Phys..

[7] Chow WW, Crawford MH (2015). Analysis of lasers as a solution to efficiency droop in solid-state lighting. Appl. Phys. Lett..

[8] Piprek J (2016). Comparative efficiency analysis of GaN-based light-emitting diodes and laser diodes. Appl. Phys. Lett..

[9] Zheng P (2019). Unique design strategy for laser-driven color converters enabling superhigh-luminance and high-directionality white light. Laser Photon. Rev..

[10] Fiederling R, Trommer J, Feil T, Hager J (2015). The next step-pure laser high-beam for front lighting. ATZ Worldw..

[11] Chi YC (2015). Phosphorous diffuser diverged blue laser diode for indoor lighting and communication. Sci. Rep..

[12] Nair GB, Swart HC, Dhoble SJ (2020). A review on the advancements in phosphor-converted light emitting diodes (pc-LEDs): Phosphor synthesis, device fabrication and characterization. Prog. Mater. Sci..

[13] Luo X, Fu X, Chen F, Zheng H (2013). Phosphor self-heating in phosphor converted light emitting diode packaging. Int. J. Heat Mass Trans..

[14] Tan CM, Singh P, Zhao W, Kuo HC (2018). Physical limitations of phosphor layer thickness and concentration for white LEDs. Sci. Rep..

[15] Cozzan C (2016). Monolithic translucent BaMgAl$$_{10O_{7}}$$: Eu $$^{2+}$$ phosphors for laser-driven solid state lighting. AIP Adv..

[16] Pust P (2014). Narrow-band red-emitting Sr[LiAl$$_3$$N$$_4$$]:Eu$$^{2+}$$ as a next-generation LED-phosphor material. Nat. Mater..

[17] Kim YH (2017). A zero-thermal-quenching phosphor. Nat. Mater..

[18] Wang L (2016). Ca$$_{1-x}$$Li$$_x$$Al$$_{1-x}$$Si$$_{1+x}$$N$$_3$$:Eu$$^{2+}$$ solid solutions as broadband, color-tunable and thermally robust red phosphors for superior color rendition white light-emitting diodes. Light Sci. Appl..

[19] Wei Y (2019). New strategy for designing orangish-red-emitting phosphor via oxygen-vacancy-induced electronic localization. Light Sci. Appl..

[20] Zhao M (2019). Emerging ultra-narrow-band cyan-emitting phosphor for white LEDs with enhanced color rendition. Light Sci. Appl..

[21] Hua Y, Yu JS (2020). Warm white emission of LaSr$$_2$$F$$_7$$:Dy$$^{3+}$$ /Eu$$^{3+}$$ NPs with excellent thermal stability for indoor illumination. J. Mater. Sci. Technol..

[22] Li S, Wang L, Hirosaki N, Xie R (2018). Color conversion materials for high-brightness laser-driven solid-state lighting. Laser Photon. Rev..

[23] Víllora EG, Arjoca S, Inomata D, Shimamura K (2016). Single-crystal phosphors for high-brightness white LEDs/LDs. Proc. SPIE.

[24] Arjoca S (2015). Temperature dependence of Ce:YAG single-crystal phosphors for high-brightness white LEDs/LDs. Mater. Res. Express.

[25] Kang TW (2017). Strong thermal stability of Lu$$_3$$Al$$_5$$O$$_{12}$$:Ce$$^{3+}$$ single crystal phosphor for laser lighting. J. Lumin..

[26] Zhang R (2014). A new-generation color converter for high-power white LED: transparent Ce$$^{3+}$$ :YAG phosphor-in-glass. Laser Photon. Rev..

[27] You S (2019). A thermally robust La$$_3$$Si$$_6$$N$$_{11}$$:Ce-in-glass film for high-brightness blue-laser-driven solid state lighting. Laser Photon. Rev..

[28] Zhang Y (2020). A high quantum efficiency CaAlSiN$$_3$$:Eu$$^{2+}$$ phosphor-in-glass with excellent optical performance for white light-emitting diodes and blue laser diodes. Chem. Eng. J..

[29] Park JY, Lee WC, Chung JW, Yang HK (2020). Phosphor-in-glass (PiG) plates for blue laser diode driven white-light emission. J. Alloy. Compd..

[30] Lin H, Hu T, Cheng Y, Chen M, Wang Y (2018). Glass ceramic phosphors: towards long-lifetime high-power white light-emitting-diode applications—A review. Laser Photon. Rev..

[31] Xiao Z (2019). Materials development and potential applications of transparent ceramics: A review. Mater. Sci. Eng. R..

[32] Song YH (2016). High power laser-driven ceramic phosphor plate for outstanding efficient white light conversion in application of automotive lighting. Sci. Rep..

[33] Yuan Y (2018). High luminous fluorescence generation using Ce:YAG transparent ceramic excited by blue laser diode. Opt. Mater. Express.

[34] Zhang Y, Hu S, Wang Z, Zhou G, Wang S (2018). Pore-existing Lu$$_3$$Al$$_5$$O$$_{12}$$: Ce ceramic phosphor: An efficient green color converter for laser light source. J. Lumin..

[35] Pricha IT, Rossner W, Moos R (2015). Pressureless sintering of Luminescent CaAlSiN$$_3$$: Eu ceramics. J. Ceram. Sci. Technol..

[36] Zhou Y, Liu F, Wang H (2017). Novel organic-inorganic composites with high thermal conductivity for electronic packaging applications: A key issue review. Polym. Compos..

[37] Chen H (2016). Thermal conductivity of polymer-based composites: Fundamentals and applications. Prog. Polym. Sci..

[38] Sichel EK, Miller RE, Abrahams MS, Buiocchi CJ (1976). Heat capacity and thermal conductivity of hexagonal pyrolytic boron nitride. Phys. Rev. B.

[39] Yuan C (2019). Modulating the thermal conductivity in hexagonal boron nitride via controlled boron isotope concentration. Commun. Phys..

[40] Lin Z (2013). Magnetic alignment of hexagonal boron nitride platelets in polymer matrix: Toward high performance anisotropic polymer composites for electronic encapsulation. ACS Appl. Mater. Interfaces.

[41] Jingyu, Z., Xiaoliang, Z., Rong, S. & Jian-bin, X. Thermal Property analysis of boron nitride-filled glass-fiber reinforced polymer composites. In *15th International Conference on Electronic Packaging Technology* 4. 10.1109/ICEPT.2014.6922655 (2014).

[42] Ishida H, Rimdusit S (1998). Very high thermal conductivity obtained by boron nitride-filled polybenzoxazine. Thermochim. Acta.

[43] Yuan C (2015). Thermal conductivity of polymer-based composites with magnetic aligned hexagonal boron nitride platelets. ACS Appl. Mater. Inter..

[44] Xie B (2018). Targeting cooling for quantum dots in white QDs-LEDs by hexagonal boron nitride platelets with electrostatic bonding. Adv. Funct. Mater..

[45] Leung SN (2013). Analytical modeling and characterization of heat transfer in thermally conductive polymer composites filled with spherical particulates. Compos. Part B Eng..

[46] Haruki M, Tada J, Tanaka K, Onishi H, Tada Y (2018). Enhancing the effective thermal conductivity of Kapton-type polyimide sheets via the use of hexagonal boron nitride. Thermochim. Acta.

[47] Nemitz W, Fulmek P, Nicolics J, Reil F, Wenzl FP (2017). On the determination of the temperature distribution within the color conversion elements of phosphor converted LEDs. Sci. Rep..

[48] Vitta P, Pobedinskas P, Zukauskas A (2007). Phosphor thermometry in white light-emitting diodes. IEEE Photon. Tech. L..

[49] Menkara, H., Summers, C., Brent, B. K. (PhosphorTech Corp) US 7109648B2 (2006).

[50] Bachmann V, Ronda C, Meijerink A (2009). Temperature quenching of yellow Ce$$^{3+}$$ Luminescence in YAG:Ce. Chem. Mater..

[51] Zhao D, Qian X, Gu X, Jajja SA, Yang R (2016). Measurement techniques for thermal conductivity and interfacial thermal conductance of bulk and thin film materials. J. Electron. Packag..

[52] de Mello JC, Wittmann HF, Friend RH (1997). An improved experimental determination of external photoluminescence quantum efficiency. Adv. Mater..

[53] Lakowicz JR (2006). Principles of Fluorescence Spectroscopy.

