# Supplementary material for: The reduction of the thermal quenching effect in laser-excited phosphor converters using highly thermally conductive hBN particles
Source: Sci Rep. 2021 Mar 24;11:6755. doi: 10.1038/s41598-021-86249-4 (PMC7991672; doi:10.1038/s41598-021-86249-4)
Supplement: Supplementary file 1 — Supplementary Information. [file 41598_2021_86249_MOESM1_ESM.pdf]

Supplementary information for

The Reduction of the Thermal Quenching Effect in Laser-Excited Phosphor Converters Using Highly Thermally Conductive hBN Particles

Akvilė Zabiliūtė-Karaliūnė, Justina Aglinskaitė, Pranciškus Vitta

Institute of Photonics and Nanotechnology, Faculty of Physics, Vilnius University, Saulėtekio al. 3, 10257 Vilnius, Lithuania

1 Measurements of the thermal conductivity

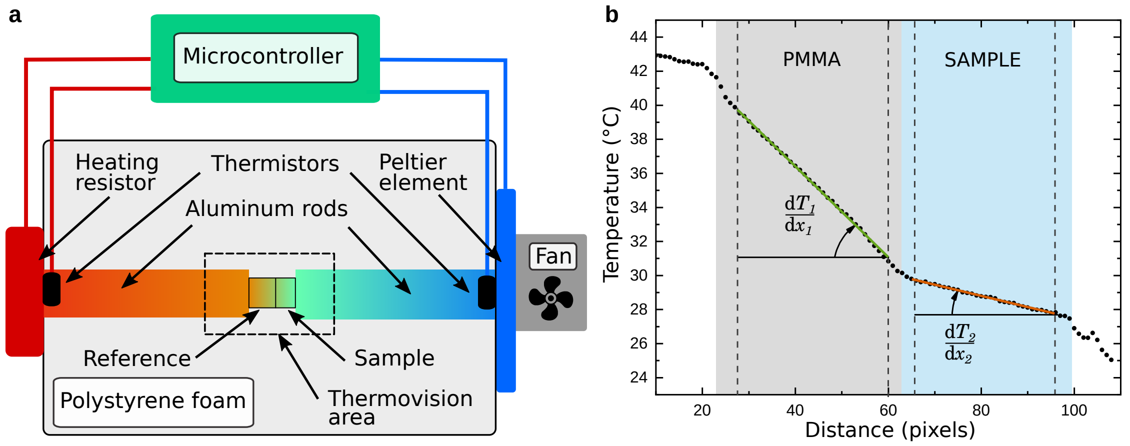

Figure S1: Measurements of TC. a) The experimental set-up for TC measurements, b) The typical gradients slopes obtained from the thermovisor. The dashed lines depict the range of data included in the slope calculation, colour lines depict linear fits.

The experimental set-up and data anlysis of TC measurements are provided in Figure S1 ab. The cylinder shaped 3 mm thickness polymethyl methacrylate (PMMA) reference and 2 mm thickness sample are placed between two aluminium rods surrounded by a thermally isolating expanded polystyrene. The outer end of one of the aluminium rods is heated with a heating resistor (red), and another is being cooled using a Peltier element (blue). The temperature is continually monitored using thermistors, that transfer data to a microcontroller controlling the heating resistor and the Peltier element. The temperature gradient of the area marked by a dashed line was recorder using a thermo-imaging device (FLIR Systems, ThermoVision A320) with a close-up lens. The longitudinal temperature dependence is depicted in Figure S2b. In order to make sure, that the change of the emissivity after loading the samples with hBN particles is not significant and does not affect the results, the calibration measurements were performed. The reference and samples with different hBN and phosphor concentrations were calibrated in a cryostat (Cryo Industries) set at 300 K temperature. The emissivity of all samples and the reference were determined to be in the range of 0.9 ± 0.03 with no observable dependence on hBN or phosphor concentration. Making an assumption that longitudinal heat flux through the system is constant and neglecting the transverse heat loses we can state that:

$$k_1 A_1 \frac{\mathrm{d}T_1}{\mathrm{d}x_1} = k_2 A_2 \frac{\mathrm{d}T_2}{\mathrm{d}x_2}, \tag{1}$$

where $k_{1,2}$ are the thermal conductivities, and $A_{1,2}$ are the cross-section areas of the reference and the sample, respectively [1]. The temperature gradients in the materials can be easily extracted from Figure S1b as slopes of the linear parts of the graph. The boundary regions are not included into the calculations. To be accurate, we have to mention that the TC and emissivity of the materials were measured only at near room temperatures ($\sim$ 300 K) and possible variation of those parameters in the range of $300 - 400$ K were not taken into account. In order to obtain the best accuracy of such experiment, the reference should be made of a hard material, with TC value close to that of a sample (in this case silicone). Since PMMA meets the above criteria (with a TC of 0.19 W m^{-1} K^{-1} given in the material datasheet) it was chosen for the production of the reference. However, PMMA was chosen as a robust reference to compare the TC of samples containing different hBN concentrations. In order to evaluate the error of the experimental set-up, the measurements were repeated for five times for each sample.

2 Frequency-domain for the determination of PL decay time

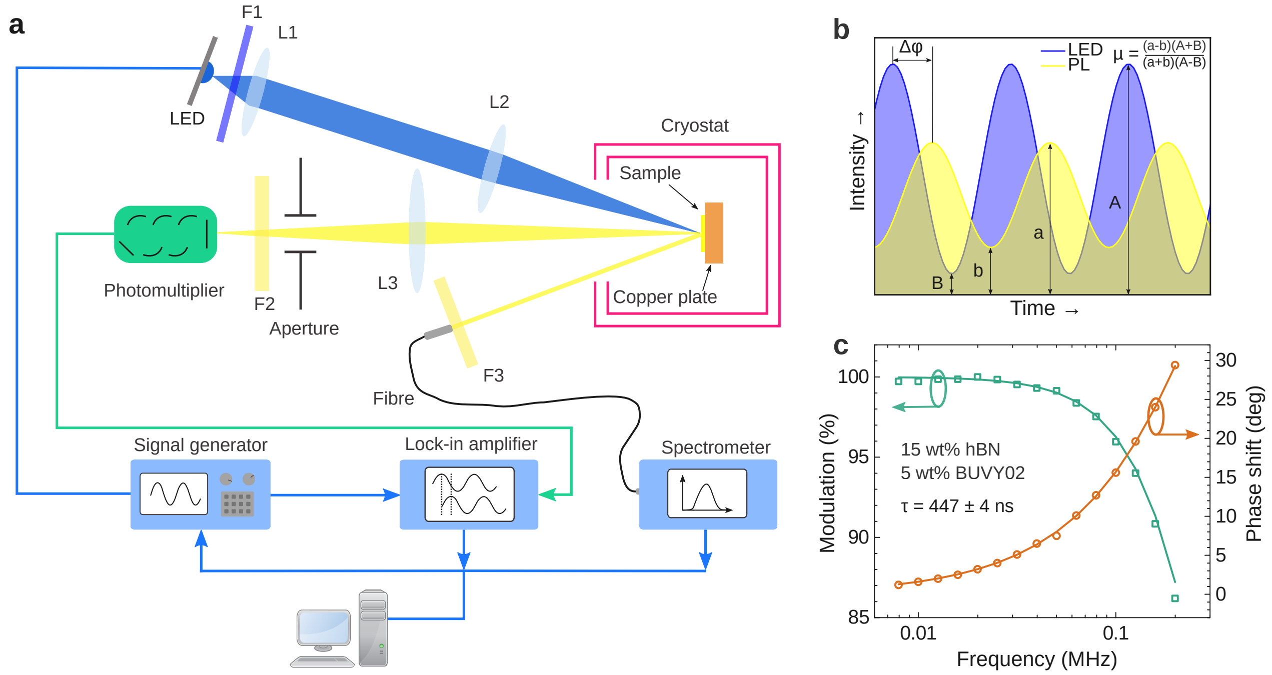

Figure S2: The FD technique fo the determination of PL decay time. a) The FD method experimental set-up. F1 is the short-pass and F2, F3 are the long-pass filters, respectively. L1, L2, and L3 are the convex lenses, used to focus the light beam. b) The illustration of the phase shift $\Delta\varphi$ between the sine-modulated excitation light (blue) and the delayed phosphor PL (yellow) as well as the modulation depth ratio μ. c) Typical dependence of the modulation depth ratio (green circles) and phase shift (orange squares) on the modulation frequency. Here, the data for the BUVY02 phosphor converter containing 5 wt% of phosphor and 15 wt% of hBN is presented.

The experimental set-up of PL decay time determination using the FD technique is presented in Figure S2a [2]. The blue LED or LD is harmonically modulated by the signal generator and focused on the sample with two lenses. As Figure S2b shows, the PL signal (yellow sine) follows the excitation (blue) with some delay due to the finite PL decay time. This results in a measurable phase shift ($\Delta\varphi$) and PL to excitation modulation depth ratio of $\mu < 1$. The phase shift and modulation depth (amplitude of the alternating component) were registered using a lock-in amplifier. The modulation depth ratio μ of PL to excitation is calculated as:

$$\mu = \frac{(a-b)(A+B)}{(a+b)(A-B)}, \tag{2}$$

where a, b, A, and B are the maximum and minimum intensity values of PL and excitation, respectively, and are shown in Figure S2. In case of a single exponential PL decay, $I(t) = I_0 \exp(-\frac{t}{\tau})$, the decay time τ can be calculated from to the following dependencies of the phase shift $\Delta\varphi$ and the modulation depth ratio μ on the modulation frequency ω:

$$\Delta\varphi = \arctan(\omega\tau); \quad \mu = (1 + \omega^2\tau^2)^{-0.5}. \tag{3}$$

The typical $\Delta\varphi(\omega)$ and $\mu(\omega)$ dependencies and their fits to the latter expressions are presented in Figure S2c. Here, 5 wt% BUVY02, 0 wt% hBN phosphor-silicone converter is excited with a blue (447 nm) LED at room temperature. The extracted PL decay time τ was found to be 447 ns.

3 Measurements of laser-excited PL decay time

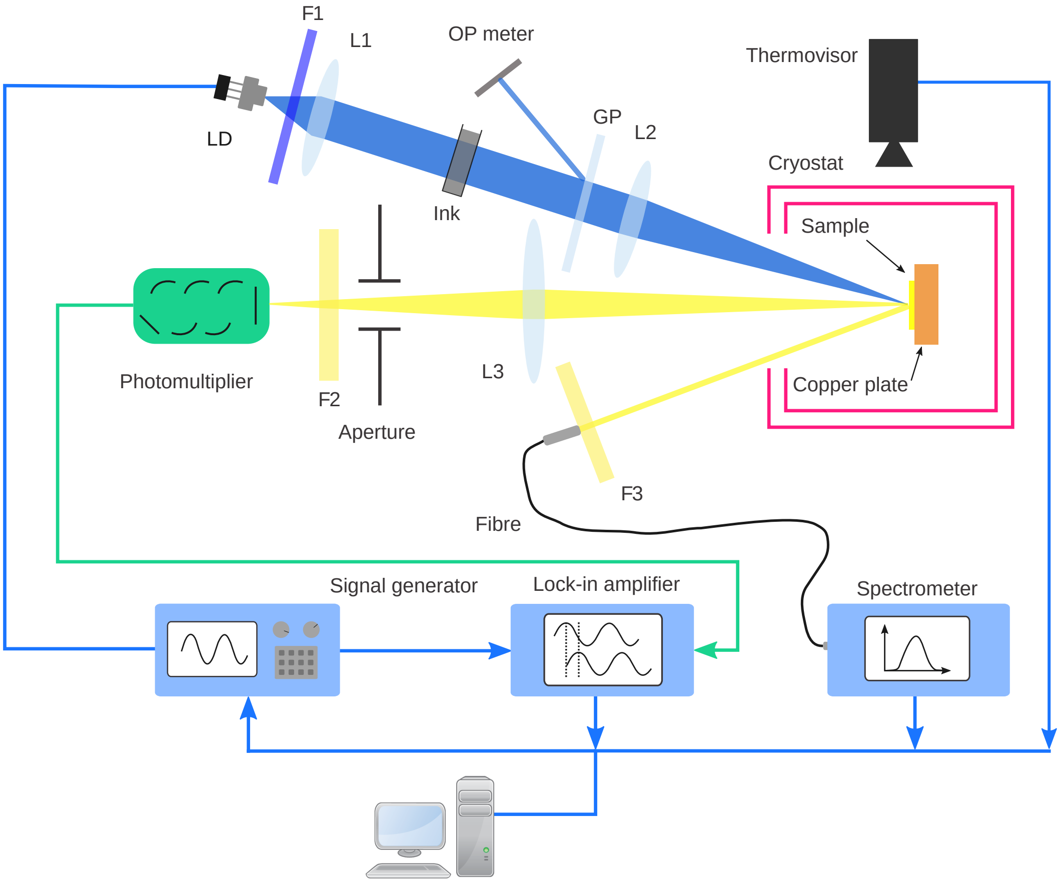

Figure S3: Experimental set-up for the measurements of laser-excited PL decay time. Here OP meter – optical power meter, GP – glass plate, L1-L3 – convex lenses, F1 – short-pass filter, F2 and F3 – long pass filters.

References

[1] Zhao, D., Qian, X., Gu, X., Jajja, S. A., and Yang, R. *J. Electron. Packaging* **138**(4), 040802 October (2016).

[2] Lakowicz, J. R. *Principles of Fluorescence Spectroscopy*. Springer, Baltimore, third edition edition, (2006).
